# Complete Genome Sequence of Human Norovirus GII.4_2006b, a Variant of Minerva 2006

**DOI:** 10.1128/genomeA.01648-15

**Published:** 2016-01-28

**Authors:** Zhihui Yang, Mark K. Mammel, Michael Kulka

**Affiliations:** Division of Molecular Biology, Office of Applied Research and Safety Assessment, Center for Food Safety and Applied Nutrition, U.S. Food and Drug Administration, Laurel, Maryland, USA

## Abstract

In 2006, the National Calicivirus Laboratory at the U.S. Centers for Disease Control and Prevention (CDC) confirmed multistate outbreaks of norovirus infection and identified two new GII.4 norovirus strains (Minerva and Laurens) through partial sequencing of the major capsid (VP1) gene. Here, we report the first complete genome sequence of the GII.4 Minerva isolate.

## GENOME ANNOUNCEMENT

Norovirus (NV) infection is recognized as the leading cause of acute viral gastroenteritis worldwide in sporadic and outbreaks cases (1). Currently, noroviruses are genetically classified into seven genogroups, which are subdivided into different genetic clusters or genotypes (2). Among the more than thirty norovirus genotypes within three human-containing genogroups (GI: genogroup I, GII: genogroup II, and GIV: genogroup IV), GII genotype 4 (GII.4) strains have been reported to cause 80% of norovirus infections worldwide (3, 4). The pandemic GII.4 variant, named GII.4 Minerva, was identified as one of two predominant outbreak strains (Minerva and Laurens) in the United States in late 2005/early 2006 (5, 6) and was sequenced to obtain the open reading frame (ORF) 2 and ORF3 coding regions (GenBank accession no. JN899246) (7). Here, we report the complete genome sequence of GII.4 Minerva associated with the 2006 outbreak of nororvirus in Ohio.

The GII.4 Minerva positive stool sample was suspended in 10% phosphate-buffered saline, centrifuged at 9000 g × 3 min, and the supernatant was filtered through a 0.22-µM membrane filter followed by viral RNA extraction using a QIAampViral RNA Mini Kit (Qiagen). Following library construction using a TruSeq stranded mRNA prep kit (Illumina) and sequencing on the MiSeq platform (Illumina), data were analyzed using CLC Genomics Workbench (CLC bio). One contig covering the full-length norovirus genome sequence was assembled from 262,715 reads containing an average coverage at 3,437×.

The complete genome sequence of GII.4 Minerva 2006 was 7,564 nucleotides (nt) in length, excluding the poly(A) tail. It contained (i) three ORFs (ORF1, ORF2, and ORF3), with lengths of 5,100, 1,623, and 807 nt, respectively, and (ii) both a 5′-untranslated region (UTR) and 3′-UTR of 5 nt and 50 nt in length, respectively. The last 3 nt likely represent the beginning of the poly(A) tail. Virus typing by phylogenetic analysis (Norovirus Genotyping tool version 1.0) confirmed that this sequence is a GII.4, and both ORF1and ORF2 nucleotide sequences subclustered Minerva as a variant of Den Haag 2006b (bootstrap value 100.0) (8). A BLAST search revealed 99% identity with multiple GII.4 Den Haag variants from Australia, Japan, Taiwan, and Korea isolated during the 2006 to 2007 period. This result supports the conclusion that closely related GII.4 2006b variants were circulating not only in the United States, but also in multiple areas worldwide in 2006 and 2007 (9–13).

The full-length GII.4 Minerva 2006 sequence was aligned with the partial sequence of Hu/GII.4/Minerva/CS1258/2006/USA (2,475 nt) available in GenBank (accession no. JN899246) and revealed a 99% homology (2,448 of 2,475 nt) with 14 nt (synonymous) differences in ORF2. Five of 13 nt differences between GII.4 Minerva and JN899246, yielding 4 amino acid differences (264 of 268), were observed in ORF3. Based on the full-length sequence, the nearest neighbor to Minerva 2006 is Hu/GII.4/Shellharbour/NSW696T/2006/AUS (GenBank accession no. EF684915.2) with 69 nt differences. Completion of the Minerva 2006 genome sequence, an important member of the GII.4 Den Haag 2006b cluster, provides an additional full-length reference sequence for phylogenetic assemblies using complete NV sequences, particularly with regard to the highly circulated Den Haag variants (14).

### Nucleotide sequence accession number.

The genome sequence of the Minerva 2006 isolate has been deposited in GenBank under the accession number KT152148.
